# Impact of Oral Environment Alterations on Salivary Candida Load: An Exploratory Clinical Study

**DOI:** 10.1055/s-0045-1812494

**Published:** 2025-11-17

**Authors:** Kamis Gaballah, Ensanya A. Abou Neel, Priyadharshini Sekar, Roba Saqan, Marwan Mansoor Mohammed, Sarah Waleed Alkhazraji, Maya Elzayat, Raneem Essam Moustafa

**Affiliations:** 1 Department of Oral and Craniofacial Health Sciences College of Dental Medicine, University of Sharjah, Sharjah, United Arab Emirates; 2 Microbiota Research Group, Research Institute for Medical and Health Sciences, University of Sharjah, Sharjah, United Arab Emirates; 3 Department of Restorative Dentistry, College of Dental Medicine, University of Sharjah, Sharjah, United Arab Emirates; 4 Biomaterials and Biomimetic Research Group, Research Institute for Medical and Health Sciences, University of Sharjah, Sharjah, United Arab Emirates; 5 UCL Eastman Dental Institute, Biomaterials and Tissue Engineering, Royal Free Hospital, Rowland Hill Street, London, United Kingdom; 6 Health Promotion Research Group, Research Institute for Medical and Health Sciences, University of Sharjah, Sharjah, United Arab Emirates

**Keywords:** *Candida*, oral environment, salivary load, dental restorations, tongue anomalies, candidal colonization, non-medicalized population

## Abstract

**Objective:**

*Candida species* are common oral commensals that can become opportunistic pathogens under specific conditions. Most research centers on medically compromised individuals, leaving a gap in understanding oral colonization patterns in healthy populations. This study aimed to investigate the impact of oral conditions and dental restorations on salivary candidal load in healthy individuals.

**Materials and Methods:**

Ninety-seven participants were divided into five groups based on the oral environment as follows: Group 1: participants with healthy mucosa and restoration-free teeth; Group 2: participants with dental fillings, conventional crowns, and fixed partial dentures; Group 3: removable dentures and appliances—positive control; Group 4: participants with tongue-coating abnormalities. Group 5: participants with partially erupted third molars. Saliva samples were collected and analyzed for *Candida* species using culture methods and colony-forming units per milliliter (CFUs/mL).

**Results:**

*Candida albicans (C. albicans)* was present in 49.5% of samples, predominantly in Group 2 (73.7%). Non-albicans *Candida* (NAC) was detected in 67% of samples, exhibiting minimal intergroup variation. Age, gender, ethnicity, plaque, and calculus scores significantly influenced the growth of *C. albicans* and NAC, with older males and Asians exhibiting higher fungal loads. Group 2 demonstrated a significant increase in *C. albicans* CFUs (10 × ) compared with the negative control. NAC load (CFUs/mL) was notably higher in participants with impacted molars. Age, oral health, and sociodemographic factors were key predictors of salivary *C. albicans* and NAC loads, with worse oral hygiene correlating to increased NAC growth.

**Conclusion:**

This study highlights the impact of the oral environment on the salivary Candidal load in disease-free populations, in particular. The presence of complex fillings, crowns, or fixed partial dentures has the most significant impact on *C. albicans* loads. However, the impacted molar has the most significant effect on NAC. The growth of both *C. albicans* and NAC was affected by the age, socioeconomic status, and oral hygiene status of the patient. Recognizing the colonization patterns in these individuals can aid in selecting restorative materials and preventive measures to reduce colonization and excessive salivary carriage. Further research is needed to explore the long-term implications of these findings on oral and systemic health.

## Introduction

*Candida* is the most common yeast isolated from the oral cavity, with up to 70% of healthy people carrying it commensally. 1 Reduced host defense or insufficient clearance has been associated with candidal colonization and opportunistic infection, leading to various oral mucosal manifestations ranging from minor denture stomatitis to potentially malignant chronic hyperplastic candidiasis. 1 2 Prolonged *Candida* colonization has been suggested to contribute to the development of oral cancer by propagating chronic inflammatory conditions and activating and generating pro-carcinogenic chemicals such as nitrosamines. 3

Specific oral settings may aid *Candida* colonization and growth. *Candida* thrives in acidic surroundings and when the host's defenses are compromised. Cigarette smoking, denture wear, xerostomia, and low saliva pH 4 5 ; and the presence of carious lesions have all been linked to more excellent *Candida* carriage rates in earlier research. 6 7 8 Candidal species' participation in early childhood caries and their synergistic relationship with *Streptococcus mutans* has offered light on prospective fungus-focused approaches to early cavitation prediction and prevention. 9

*Candida* carriage rates have been measured more frequently in medically impaired groups than in the general population. Assessing *Candida* carriage risk factors in a “non-medicalized” population could be helpful in future oral health and cancer screening regimens. To date, no investigation has been conducted to determine the prevalence and severity of salivary candidal carriage in patients with tongue deformities such as benign migratory glossitis, fissured tongue, or hairy tongue. The influence of having dental restorations other than removable dentures was not looked at.

The existence of a partially erupted lower third molar is a common oral health finding, and there is no standardized treatment approach in the literature for removing or preserving such teeth over an extended time. Many clinicians choose to retain such teeth for patient safety and to avoid the potential complications of surgical procedures. However, some data suggest that these teeth may negatively affect patients' oral and general health. 10 The operculum covers the partially erupted third molar teeth, allowing food debris to accumulate and potentially providing a safe environment for diverse oral microbial flora growth. This may influence the increase in salivary carriage of oral bacteria and, possibly, candidal species. Suppose this hypothesis is not rejected through research. In that case, it may shed light on the impact of third molars on general dental health and partially account for the retained third molar's participation in the rise of systemic inflammation markers.

Furthermore, materials like polymeric denture bases (polymethyl methacrylate [PMMA]), resin composites, and ceramics are commonly used as permanent restorations that will be used for several years. Dental appliances such as removable complete and partial dentures have been shown to promote *Candida albicans* ( *C. albicans* ) growth and cause mucosal diseases. 11 12

*Candida* is an endogenous commensal organism that can transition into a pathogenic state under certain conditions. Unlike many infectious diseases, where the organism's virulence factors primarily drive the pathogenicity, *Candida* infections are often associated with host-related or environmental factors. This highlights the strictly opportunistic nature of *Candida* species, as they cause disease only when the host's defense mechanisms are compromised or the environment becomes favorable. There are numerous species of *Candida* , 13 but the most frequently isolated species from the oral cavity, both in its commensal form and in cases of oral candidiasis, is *C. albicans* . This species is estimated to account for over 80% of all oral yeast isolates. In addition to *C. albicans* , there has been increasing recognition of the significance of *non-albicans Candida* (NAC) species in human disease. *C. glabrata* and *C. krusei* have garnered particular attention due to their increased resistance to certain antifungal agents. Another species whose presence is clinically significant is *C. auris* . 14

This study, therefore, aims to investigate (1) the impact of oral conditions and dental restorations on salivary candidal load in healthy individuals by measuring the candidal load of individuals with normal oral mucosa, restoration-free dentition or appliances, and those with various restorations or appliances, and (2)the effect of tongue surface coating anomalies and presence of partially erupted mandibular third molar teeth on the candidal load in saliva.

## Materials and Methods

### Ethical Approval and Study Design

This prospective experimental study was conducted at the College of Dental Medicine and the Microbiota Research Laboratory, RIMHS, University of Sharjah, in the United Arab Emirates. The present study was conducted in accordance with the ethical guidelines outlined in the World Medical Association's Declaration of Helsinki, which governs medical research involving human subjects. All participants were provided with both verbal and written information about the study and the potential use of their anonymized medical data for research purposes. Their consent was obtained using an institutionally approved consent form. Furthermore, all participants were informed of their right to withdraw from the study at any time. The research was granted permission by the University of Sharjah Research Ethics Committee (approval number: REC-22-02-10-01). All participants were informed in detail about the study's aims and steps and were asked to sign an informed consent form.

### Study Participants

This study focused on adult patients with permanent teeth, specifically those between 15 and 50 years old. Although the age group of participants ranged from 15 to 50, all participants were aged 18 years and above, so consent from parents or legal guardians was not required. Patients with mixed teeth or those who have diabetes, anemia, or who take steroids, antifungal medications, or antibiotics in the last 3 months were excluded from the study. Intraorally, patients with clinical evidence of periodontal disease or overt oral mucosal inflammation were excluded. Additionally, participants with oral and dental findings that overlapped between groups were not included. Patients with mixed types of restoration and/or appliances were also excluded. Detailed information about each patient, including name, age, gender, ethnicity, contact number, medical and drug history, and oral examination findings, was collected. Furthermore, the oral health status and conditions of the participants were also collected. The study manuscript does not include any personal or clinical details that would compromise participant anonymity. Therefore, consent for publication was deemed unnecessary.

### Collection and Processing of Saliva Samples for Enumerating Candidal Load

For collecting and measuring salivary candidal load, patients were requested not to eat for at least one hour before sampling. Patients were then instructed to thoroughly rinse their mouth with 20 mL of sterile normal saline for 2 minutes. The rinse was then collected and centrifuged at 3,500 rpm for 15 minutes. After centrifugation, the resulting pellets were reconstituted in 1 mL of phosphate-buffered saline (PBS) (Dulbecco's PBS, Sigma D8537) to obtain a uniform suspension. Ten µL and 100 µL of this suspension were plated by lawn culture onto *Candida* Chromogenic Agar, Condalab (Cat. 1382), then incubated at 37 °C aerobically for 24 hours. The *Candida* identification and speciation were done based on the color of the colonies. The presence of green-colored colonies indicated the growth of *C. albicans* , blue-colored colonies indicated the growth of *C. tropicalis* , white-colored colonies with brown centers indicated *C. glabrata* , and purple-pink colonies indicated the growth of *C. auris* or *C. krusei* . Colonies of *C. albicans* were counted and calculated for CFU/mL and recorded as *C. albicans* CFU/mL. Colonies of all other *Candida* species were counted and calculated for CFU/mL and recorded as NAC.

### Statistical Analysis

The data was analyzed using SPSS (Statistical Package for the Social Sciences), version 22, by IBM Corp., United States. Descriptive statistics for continuous data were presented as mean ± standard deviation or median (interquartile range [IQR]), depending on data normality, and for categorical data as frequencies and percentages. The chi-square test was used to examine differences in *Candida* distribution across various oral condition groups. Similarly, the Kruskal–Wallis test was employed to investigate the difference in *Candida* load across various oral condition groups. Simple and multiple logistic regression analyses were conducted to identify factors influencing the presence of *Candida* . Additionally, zero-inflated Poisson regression was employed to investigate the factors influencing the *Candida* load within the sample. A *p* -value of less than 0.05 was considered statistically significant.

## Results

### Sociodemographic Analysis of Participants

The total number of participants who were interviewed, examined, and donated a salivary sample for testing was 97 out of 100 who accepted to take part in the study. The age of the participants ranged from 18 to 60 years, with a mean age of 33.2 years. Most participants were between the ages of 18 and 25, followed by nearly equal numbers in the 26- to 35-year-old and 36- to 45-year-old age groups. The study sample was equally distributed among both genders. The ethnicity data revealed an equal distribution between Arab and Asian ethnicities ( Table 1 ).

**Table 1:** Descriptive statistics and oral Health status and conditions of the participants ( *n* = 97)

Sociodemographic		
		Frequency (%)
Age groups	15–25	51 (52.58)
	26–35	19 (19.59)
	36–45	17 (17.53)
	>45	10 (10.31)
*Overall age, mean ± SD = 33.17 (11.61)*		
Gender	Male	49 (50.5)
	Female	48 (49.5)
Ethnicity	Arabs	53 (54.6)
	Asian	44 (45.5)
**Oral hygiene status**		
Plaque score	0	4 (4.1)
	1	73 (75.3)
	2	16 (16.5)
	3	4 (4.1)
*Median (IQR) = 1 (0.25)*		
Calculus score	0	8 (8.2)
	1	59 (60.8)
	2	14 (14.4)
	3	13 (13.4)
	4	3 (3.1)
*Median (IQR) = 1 (0)*		
**Oral health conditions**		
Group 1	Healthy mouth	17 (100)
Group 2	Composite	9 (47.4)
	Onlays	1 (5.3)
	Crowns	5 (26.3)
	Bridge	4 (21.1)
Group 3	RPD	7 (36.8)
	Orthodontic	7 (36.8)
	Full denture	5 (26.3)
Group 4	Fissured 25%	5 (25.0)
	Fissured 50%	4 (20.0)
	Fissured 75%	4 (20.0)
	Geographic 50%	5 (25.0)
	Hairy tongue	2 (10.0)
Group 5	Unilateral impaction	16 (72.70)
	Bilateral impaction	6 (27.30)

Abbreviations: IQR, interquartile range; SD, standard deviation.

Notes: Categorical variables are reported as frequencies (%); continuous variables are reported as mean ± SD and median (IQR) for those not following a normal distribution.

Descriptive statistics of sociodemographic data and oral hygiene status and conditions of the participants ( *n* = 97).

### Oral Health of the Study Participants

The oral health status of the participants was determined by dental plaque and calculus scores. This was graded on a scale of 0 to 3 and 0 to 4 for plaque and calculus, respectively. The dental plaque score was pooled around the value of 1. Similar observations were noted with the dental calculus score ( Table 1 ).

### Grouping of Study Participants Based on Oral Health Conditions

Based on their oral health condition, the participants were divided into four groups ( Table 1 ):

Group 1: Seventeen participants with healthy mouths, showing no clinically detectable oral or dental diseases and having teeth and mouths free of restorations and appliances, were assigned to this group. This group served as the negative control.

Group 2: Nineteen participants with more than two dental fillings involving multiple surfaces, conventional crowns, fixed partial dentures, or implant-supported crowns were assigned to this group.

Group 3: Nineteen participants who had removable appliances, including full and partial dentures and orthodontic removable appliances, were assigned to this group, and these served as the positive control.

Group 4: Twenty participants who had tongue coating abnormalities, including benign migratory glossitis, hairy tongue involving more than one-third of the dorsal surface, median rhomboid glossitis, and fissured tongue involving more than one-third of the tongue, were assigned to this group.

Group 5: This group consisted of 22 participants with partially erupted lower third molar teeth. Among these individuals, 16 had unilateral impacted third molars, while 6 had bilateral impacted third molars.

### Salivary Candidal Carriage and Statistical Analysis

*C. albicans* from the saliva samples of the study participants was identified and differentiated from NAC using *Candida* Chromogenic Agar. CFU/mL was calculated from the number of colonies observed on the culture plate. Data were recorded and analyzed statistically by comparing the presence of *C. albican* s and NAC with the different oral conditions, oral health, and sociodemographic details of the study participants.

### Frequency and Load (CFU/mL) of *C. albicans* and *Non-albicans Candida* Across Different Oral Conditions

The saliva sampled from all groups yielded *C. albicans* growth at variable rates. The lowest growth rate was recorded for samples from Group 1 participants at a frequency of 23.5% (IQR: 2,207.5 CFU/mL), while the maximum growth was recorded for samples from Group 2 participants at a frequency of 73.7% (IQR: 3,428.8 CFU/mL). There were statistically significant differences between the groups ( *p* = 0.002). On the other hand, the NAC was detected in most samples across the groups, with a narrow margin of frequency, ranging from 54.5% (IQR: 2,298.8 CFU/mL) to 78.9% (IQR: 130 CFU/mL), in samples taken from participants in groups 5 and 3. Overall, the frequency of *C. albicans* spp. was observed in half of the samples ( *n* = 48; 49.5%), and 65 samples (67%) yielded salivary NAC ( Table 2 ).

**Table 2:** The *Candida* status, both frequency ( *N* %) and load (CFU/mL) across each group

		Presence of Candida Frequency (%)			Load (CFU/mL)	
		No	Yes	*p* -Value	Median (IQR)	*p* -Value
**Salivary** ***C. albicans***						
Groups	1	13 (76.5)	4 (23.5)	0.002 a	110 (2,207.5)	0.766
	2	5 (26.3)	14 (73.7)		135 (3,428.8)	
	3	9 (47.4)	10 (52.6)		1,400 (2,465)	
	4	6 (30.0)	14 (70.0)		475 (3,011.3)	
	5	16 (72.7)	6 (27.3)		357.5 (538.8)	
**Salivary NAC**						
Groups	1	7 (41.2)	10 (58.8)	0.441	50 (45)	0.712
	2	5 (26.3)	14 (73.7)		250 (285)	
	3	4 (21.1)	15 (78.9)		100 (130)	
	4	6 (30.0)	14 (70.0)		105 (546.3)	
	5	10 (45.5)	12 (54.5)		155 (2,298.8)	

Abbreviation: IQR, interquartile range.

Notes: “Yes” indicates the presence of *Candida* , while “No” indicates the lack of *Candida* in salivary samples.

Categorical variables are reported as frequencies (%); continuous variables are reported as median (IQR).

The *p* -values were derived using the chi-square test for categorical variables and the Kruskal–Wallis test for continuous variables.

Frequency (%) and load (CFU/mL) of salivary *C. albicans and* salivary *NAC* across groups.

a Significance.

### Factors Affecting the Presence of Salivary *C. albicans* and Salivary *Non-albicans*

An adjusted logistic regression model was used to examine factors affecting the presence of salivary *C. albicans* and NAC. The relationship between the age of the participants and the number of samples yielded NAC growth was found to be proportional. The younger participants showed the least growth, while the older ones exhibited more growth, with a clear statistical significance between them ( *p* = 0.001). On the other hand, the sample proportional relationship was also noted with the growth of the *C. albicans* culture. However, no statistical significance was reported ( *p* = 0.195).

Regarding the effect of gender, there was a tendency for more significant growth in salivary samples from males compared with those from females. Still, there were no significant differences between the two genders. The same pattern was observed in terms of ethnicity, where salivary samples from Asian participants yielded more candidal growth than those from Arab participants, but again, no statistical significance was reported between the two groups.

Regarding the effect of plaque and calculus scores, the number of samples that yielded growth of both *C. albicans* and NAC was related to the score of both plaque and calculus deposits obtained from the participants' dental examination. The growth was likely associated with higher plaque and calculus scores. However, statistical significance was only related to the NAC growth, which was particularly associated with the higher plaque indices ( Table 3 ).

**Table 3:** Factors affecting the presence of salivary *C. albicans* and salivary NAC (yes, no)

		Salivary *albicans*			Salivary NAC		
		OR	95% CI	*p* -value	OR	95% CI	*p* -value
Groups	Ref: 1						
	2	9.7	[1.71, 55.20]	0.010 a	0.6	[0.10, 3.42]	0.562
	3	3.52	[0.61, 20.39]	0.161	0.91	[0.15, 5.45]	0.921
	4	6.74	[1.31, 34.70]	0.023 a	0.76	[0.16, 3.61]	0.730
	5	1.04	[0.21, 5.14]	0.960	0.59	[0.14, 2.53]	0.479
Age groups	Ref: 15–25						
	26–35	1.08	[0.30, 3.91]	0.909	4.86	[1.23, 19.14]	0.024 a
	36–45	0.72	[0.14, 3.72]	0.697	9.12	[1.29, 64.58]	0.027 a
	> 45	0.62	[0.09, 4.49]	0.640			
Gender	Ref: Female						
	Male	1.54	[0.55, 4.29]	0.412	1.28	[0.43, 3.76]	0.655
Ethnicity	Ref: Arabs						
	Asian	2.64	[0.84, 8.32]	0.097	0.7	[0.21, 2.38]	0.571
Oral hygiene status	Plaque Score	0.98	[0.30, 3.15]	0.971	1.87	[0.53, 6.52]	0.328
	Calculus Score	0.85	[0.41, 1.78]	0.668	0.88	[0.42, 1.86]	0.736

Notes: Adjusted logistic regression module for the following variables: oral conditions, age, gender, ethnicity, plaque, and calculus.

Ref: means the reference group for calculation of OR.

Odd ratio (OR), 95% confidence interval (CI), and *p* -value of salivary *C. albicans* and salivary NAC for the study groups, age groups, gender, ethnicity, plaque, and calculus scores.

a Significance.

### Distribution of Oral Findings and the Presence of Salivary *C. albicans* and Salivary *Non-* albicans across Different Sub-groups

Regarding the impact of different dental restorations on the salivary candidal load, all samples collected from mouths containing crowns and onlays (Group 2) yielded 100% of both *C. albicans* and NAC. However, only 50% of patients with bridges had *C. albicans* , while 100% of the patients had NAC. Of the salivary samples from participants with resin composites, 66.7% had *C. albicans* , whereas 44.4% had NAC. For Group 3, *C. albicans* was observed in 57.1 and 60% of RPDs and full dentures, respectively, whereas 100% of NACs were observed in both RPDs and full dentures.

On the other hand, using orthodontic appliances resulted in only 42.9% of both *C. albicans* and NAC growth. For Group 4, the presence of 25% fissuring of the dorsal surface of the tongue resulted in 40 and 60% of *C. albicans* and NAC growth, respectively, while the tongue with 50% fissuring yielded 100% of both *C. albicans* and NAC growth and the sample from participants with 75% fissured tongue resulted in only 50% of both *C. albicans* and NAC growth. The number of samples yielded growth increased with the tongue's surface area. Benign migratory glossitis (geographic tongue) was related to 80 and 60% of the salivary samples for *C. albicans* and NAC spp., respectively. All salivary samples taken from patients with hairy tongues yielded the growth of all tested *Candida* spp. Concerning Group 5, *C. albicans* was isolated from 25% of the samples collected from participants with unilateral, partially impacted third molars and 33.3% of the samples from those with bilateral, partially impacted third molars. Conversely, NAC growth was identified in a significantly higher proportion of participants, with 56.3% of samples from individuals with unilateral partially impacted third molars and 50% from those with bilateral partially impacted third molars (see Table 4 ).

**Table 4:** The distribution of oral findings and the presence of salivary *C. albicans* and salivary NAC across different sub-groups

		Oral findings	Salivary *albicans*		Salivary NAC	
			Frequency (%)		Frequency (%)	
			No	Yes	No	Yes
Groups	1	Healthy mouth	13 (76.5)	4 (23.5)	7 (41.2)	10 (58.8)
	2	Composite	3 (33.3)	6 (66.7)	5 (55.6)	4 (44.4)
		Onlays	0 (0)	1 (100)	0 (0)	1 (100)
		Crowns	0 (0)	5 (100)	0 (0)	5 (100)
		Bridge	2 (50)	2 (50)	0 (0)	4 (100)
	3	RPD	3 (42.9)	4 (57.1)	0 (0)	7 (100)
		Orthodontic	4 (57.1)	3 (42.9)	4 (57.1)	3 (42.9)
		Full Denture	2 (40)	3 (60)	0 (0)	5 (100)
	4	Fissured 25%	3 (60)	2 (40)	2 (40)	3 (60)
		Fissured 50%	0 (0)	4 (100)	0 (0)	4 (100)
		Fissured 75%	2 (50)	2 (50)	2 (50)	2 (50)
		Geographic	1 (20)	4 (80)	2 (40)	3 (60)
		Hairy tongue	0 (0)	2 (100)	0 (0)	2 (100)
	5	Unilateral impaction	12 (75)	4 (25)	7 (43.8)	9 (56.3)
		Bilateral impaction	4 (66.7)	2 (33.3)	3 (50)	3 (50)
*p* -Value			0.035 a		0.113	

Notes: The *p* -values were derived using the chi-square test for categorical variables. “Yes” indicates the presence of *Candida* , while “No” indicates the lack of *Candida* in salivary samples.

Frequency (%) of presence or absence of salivary *C. albicans* and salivary NAC for the study groups.

a Significance.

### Candidal Colony-Forming Units across Different Groups

Table 5 presents a descriptive analysis of CFU counts across different groups. Almost all groups showed higher mean counts of CFUs than those reported for Group 1 (control). Despite the lack of clear statistical significance between the various groups, there were noticeable differences between them compared with Group 1. These differences were particularly evident in the *C. albicans* CFU count. When compared with Group 1's CFU count, Group 2 exhibited more than a significant increase (10 × ), while Groups 3 and 4 exhibited more than a 7× and 5× increase, respectively. The NAC CFUs were doubled only in Groups 2, 3, and 4 as compared with Group 1. However, the NAC CFU mean in Group 5 was 11× higher than that reported for Group 1. In contrast, Group 5 (the partially erupted third molar) showed no increase in CFUs of *C. albicans* ( Table 5 ).

**Table 5:** Descriptive analysis of CFU counts across different groups, highlighting fold differences

	*C. albicans* CFUs				Fold changes	NAC CFUs			Fold changes
		Mean	SD	Difference		Mean	SD	Difference	
Groups	1	192.35	721.96	0.00	1.00	81.76	211.87	0.00	1.00
	2	1,994.21	4,099.11	1,801.86	10.37	165.26	192.28	83.50	2.02
	3	1,387.00	3,511.05	1,194.65	7.21	176.32	340.09	94.55	2.16
	4	1,049.75	1,843.58	857.40	5.46	245.75	405.42	163.99	3.01
	5	87.27	200.38	−105.08	0.45	908.41	2,382.06	826.64	11.11

Note: Descriptive analysis of CFU counts across different groups, highlighting fold changes while considering group 1 as a control.

### Influence of Sociodemographic and Oral Health on Candidal Load

Zero-inflated Poisson regression was used to explore the determinants affecting the salivary *C. albicans* load. The presented findings reveal that with each unit increase in age, there is a corresponding 1.01 increase in the salivary *C. albicans* load. Likewise, for every unit increase in Plaque and Calculus scores, there is a 0.82 and 2.20 rise in the salivary *albicans* load, respectively. Males exhibited a 0.45 likelihood of having salivary *albicans* compared with females, while Asians are 0.84 times more likely to have a load than Arabs. The salivary *C. albicans* load is declining across different groups. The highest load in Group 2 is 2.74 times greater than in Group 1, whereas in Group 5, it is only 0.12 times the load observed in Group 1. Overall, the results suggested that group type, age, gender, ethnic group, plaque, and calculus are all significant predictors of the salivary *C. albicans* load.

Zero-inflated Poisson regression was also employed to examine the factors affecting the load of salivary NAC. The output suggested that the salivary NAC load for males is 0.64 times that for females. Asians have a salivary NAC load 5.12 times higher than Arabs. Additionally, the analysis indicated that with each increment in age, the load has a corresponding increase of 0.92. Similarly, for every unit increase in Plaque and Calculus scores, there is a rise of 1.69 and 1.67, respectively, in the salivary NAC load. Compared with Group 1, the salivary NAC load is 3.76 times higher in Group 5, 2.08 times higher in Group 3, 1.36 times higher in Group 4, and 1.20 times higher in Group 2. The results suggested that the group type, age, gender, ethnic groups, plaque, and calculus are all significant predictors of salivary NAC load ( Table 6 ).

**Table 6:** Factors affecting the salivary *C. albicans* load and the salivary NAC load (CFU/mL) ( *n* = 97)

		Salivary *C. albicans*				Salivary NAC	
		OR	95% CI	*p* -Value	OR	95% CI	*p* -Value
Groups							
	Ref [1]						
	2	2.74	[2.64, 2.85]	<0.001	1.20	[1.12, 1.29]	<0.001
	3	2.62	[2.52, 2.73]	<0.001	2.08	[1.94, 2.24]	<0.001
	4	1.90	[1.83, 1.98]	<0.001	1.36	[1.27, 1.45]	<0.001
	5	0.12	[0.12, 0.13]	<0.001	3.76	[3.55, 3.99]	<0.001
Age		1.01	[1.01, 1.01]	<0.001	0.92	[0.92, 0.92]	<0.001
Gender							
	Ref [Female]						
	Male	0.45	[0.44, 0.45]	<0.001	0.64	[0.62, 0.65]	<0.001
Ethnicity							
	Ref [Arabs]						
	Asian	0.84	[0.82, 0.85]	<0.001	5.12	[4.97, 5.27]	<0.001
Oral hygiene status	Plaque	0.82	[0.81, 0.84]	<0.001	1.69	[1.64, 1.74]	<0.001
	Calculus	2.20	[2.18, 2.22]	<0.001	1.67	[1.64, 1.71]	<0.001

Notes: Odd ratio (OR), 95% confidence interval (CI), and *p* -value of CFUs/mL of salivary *C. albicans* and salivary NAC for the study groups, age groups, gender, ethnicity, plaque, and calculus scores.

## Discussion

This study aimed to explore salivary candidal carriage among individuals with various oral conditions and dental rehabilitative procedures using multiple materials. The hypothesis was that changes in the oral environment would likely favor the enrichment of *C. albicans* and pathogenic NAC in the oral cavity, thereby affecting its presence in the saliva. The importance of this study lies in its potential to answer why some patients may have vague oral mucosal symptoms without detectable mucosal candidal lesions. This might be related to the high concentration of *Candida* with their toxins in those individuals' saliva, which might be triggered by the alteration of teeth or the tissue using the various dental restorations and appliances, or other abnormalities in the oral mucosa that are usually not regarded as pathological. Examples of these conditions include multiple forms of tongue coating conditions or the presence of clinically asymptomatic impacted lower third molar teeth. Additionally, with mounting evidence suggesting that *Candida* may play a role in caries and periodontal disease, it is crucial to maintain the salivary candidal load at a controlled level to reduce the future burden of these diseases.

The results of this study confirmed, to varying extents, that changes in oral environments significantly impacted the presence of detectable candidal loads isolated from the salivary samples of participants. Overall, around half of the collected samples yielded candidal growth, a figure comparable to that obtained from an equal sample size by Alrayyes et al and Darwazeh et al. 15 16 The exact rate was also reported in more than 200 individuals by Mun et al. 17 All these reports were performed on participants free from clinically detectable oral candidal lesions. It is not uncommon to have a relatively high salivary candidal load in asymptomatic individuals without any debilitating systemic condition like diabetes, HIV, immunosuppressive cytotoxic medications, or radiation to the head and neck region.

The number of samples showing candidal growth was related to the age of the participants. A younger age was associated with fewer positive samples, while an older age was linked to more positive samples. This relationship was statistically significant ( *p* < 0.001) for the NAC spp. A similar relationship was observed with the growth of the *albicans* culture, but it did not show statistical significance ( *p* = 0.195). Similar outcomes were reported by Darwazeh et al and Al-Amad et al. 6 18 The increase in salivary carriage with age may be correlated with xerostomia, which is more prevalent with age, as well as with changes in the oral environment associated with tooth loss and the use of various dental prostheses that can harbor more *C. albicans* . This increase in salivary carriage with age may also be related to the presence of periodontal pockets, where these microbes can thrive in a protected, humid environment. 19

Our results showed a tendency for growth to be related to higher plaque and calculus scores, highlighting the role of oral hygiene in shaping the oral microbial ecosystem. Poor oral hygiene, as reflected in increased plaque accumulation and calculus formation, creates a biofilm-rich environment with reduced oxygen tension and increased availability of fermentable substrates, conditions that facilitate *Candida* colonization and persistence. This supports the notion that *Candida* is involved in early caries activity in young patients. 6 The evident association between candidal colonization in carious cavities has led some investigators to consider *Candida* a key pathogen in dental caries. 20

Similarly, higher plaque and calculus scores are common features of periodontal disease, which aligns with previous reports showing that patients with periodontal disease harbor higher levels of *Candida* spp. 21 22 In fact, some studies suggest that *Candida* spp. may contribute to a more severe course of periodontitis through synergistic interactions with periodontal pathogens. 21 23

Oral hygiene practices also appear to influence *Candida* carriage. For example, the use of mouthwash, particularly non-alcohol preparations, has been associated with reduced salivary candidal levels in individuals without fungal mucosal pathologies. Although not statistically significant in our study, alcohol-based mouthwashes were less effective in lowering salivary candidal levels. 17 Collectively, these findings underscore that maintaining adequate oral hygiene—by controlling plaque and calculus accumulation—is an essential preventive measure not only against caries and periodontal diseases but also against excessive candidal colonization.

This study's findings underscore the role of various dental restorations, oral appliances, and oral conditions in shaping candidal colonization patterns, particularly differentiating the behavior of *C. albicans* and NAC species. In Group 2, where all samples from patients with crowns, bridges, and onlays yielded candidal growth, the results align with existing literature highlighting dental prostheses as key contributors to increased oral candidal load. Studies by Ramage et al and Sardi et al suggested that the surface roughness, porosity, and biofilm-forming potential of these materials create favorable environments for candidal adhesion and proliferation. 24 25 Interestingly, the 50% candidal growth observed in patients with composite resin restorations may point to the antimicrobial properties of certain resin materials or differences in oral hygiene practices associated with these restorations. 26 In the existing literature, a significant focus has been on removing dentures as a strategy to alleviate denture stomatitis and mitigate the elevated salivary candidal load. 12 27 28 Less emphasis was given to other restorations. However, several attempts have been made to investigate candidal adhesion to various materials in vitro. 29 30 31 For Group 3, involving patients with full or partial dentures, the consistent growth of NAC species and the lower incidence of *C. albicans* growth contrast with some earlier studies that identified *C. albicans* as the predominant species in denture wearers. 32 This discrepancy may be due to differences in study populations, denture hygiene practices, or the microbial environment under prostheses, which can vary based on factors such as saliva flow and denture material. 33 34 35 The lower candidal growth associated with orthodontic appliances (42.9%) suggests that these devices' physical and material properties may not support candidal colonization as extensively as other prostheses. 36

In an ongoing study, we observed that the candidal load varies according to the material from which the restoration is made, as well as the material properties such as roughness and contact angle. As observed, both dental amalgam and resin composites showed higher candidal load than other tested materials (E-max, zirconia, PMMA, cobalt-chromium) (Gaballah K et al, unpublished data). Furthermore, *C. albicans* can be pathogenic under certain circumstances. For example, exposing the polymethylmethacrylate surface to cyclic strain transforms *C. albicans* into a pathogenic form as evidenced by increasing the formation of biofilm mass, expression of virulent factors (pseudo-hyphae cells), genes (UME6 and HGC1), and enzymes (LIP, PLB, and SAP families). This underscores the importance of treating any fungal infection associated with biomaterials such as dentures and prostheses. 37 Furthermore, the surface characteristics of a material have a great impact on candidal loading as well. 38 Studying candidal load on different surfaces provides information that helps in the selection of the appropriate material, particularly for those with medically compromised individuals. 29

Group 4's analysis of oral mucosal conditions revealed significant associations with candidal growth. The high prevalence of candidal growth in patients with a fissured tongue supports the hypothesis that the deep grooves provide protected niches for fungal colonization. 39 40 The high candidal growth associated with benign migratory glossitis further emphasizes the role of altered mucosal environments in promoting fungal colonization, consistent with findings by Bánóczy et al. 41 The universal candidal growth in patients with hairy tongues observed in this study mirrors results from Joseph and Savage, 42 indicating that the keratinized papillae create an optimal environment for fungal adherence. 39 40

In Group 5, the differential candidal growth patterns observed in patients with partially impacted third molars showed that NAC species were more prevalent than *C. albicans* , suggesting a unique ecological niche associated with this condition. The presence of partially erupted third molars has been linked to systemic inflammation and may potentially affect patients' general health. Individuals with impacted third molars have shown elevated levels of C-reactive protein and interleukin-6 compared with those without impacted third molars. 10 Furthermore, research suggests that young pregnant females with third molars may be more prone to developing periodontitis, leading to a heightened level of systemic inflammation. 43 Considering these observations with the present findings, the increase in candidal colonization may play a role in the reported local and systemic inflammation. The lower *C. albicans* CFUs in this group may be attributed to localized inflammatory conditions that favor NAC species. However, a similar finding was reported following radiation after cancer resection 44 ; this hypothesis requires further investigation. 44 The surface roughness and micro-porosities of restorative materials, despite the finishing, provide ideal niches for biofilm formation. *C. albicans* excels in colonizing such surfaces by forming strong adhesins and sturdy biofilms. Furthermore, the supragingival and readily accessible nature of these restorations may create a more aerobic environment, which is favorable for *C. albicans* compared with some NAC species. The microbial ecosystem surrounding these restorations may also be influenced by constant sugar availability, promoting a cariogenic biofilm in which *C. albicans* is known to synergize with bacteria such as *Streptococcus mutans* . 6 Conversely, the partially erupted third molar environment grants a distinct ecological niche that may favor NAC. The pericoronal pocket is often deep, anaerobic, and inflamed, characterized by a low redox potential. Such an anaerobic milieu may be less conducive to *C. albicans* and more favorable for NAC species. Anaerobic periodontal pathogens likely dominate the biofilm structure in such subgingival and complex areas. This altered microbial community, along with the host's inflammatory response, may create a competitive environment that selectively promotes the growth of NAC over *C. albicans* . 45

Though lacking statistical significance, the quantitative analysis of CFUs across the groups revealed substantial trends. The marked increase in *C. albicans* CFUs in Groups 2 and 3 compared with the control group (Group 1) underscores the significant impact of dental restorations and appliances on candidal load, corroborating findings by Scully et al 13 and Samaranayake et al. 5 However, the more modest increase in NAC CFUs, particularly the significant rise in Group 5, contrasts with these studies and suggests that NAC species may respond differently to the oral environment created by specific conditions.

The regression analysis provided additional insights into the predictors of salivary candidal load. The positive association between age, plaque, and calculus with both *C. albicans* and NAC loads is consistent with the literature, which identifies these factors as critical in promoting fungal colonization. 46 The observed gender and ethnic differences in candidal load highlight the need for a personalized approach to managing oral candidiasis, as demographic factors appear to influence fungal load significantly, corroborating findings by Arendorf and Walker 1 and Xiao et al. 47

This study investigated salivary candidal carriage among healthy individuals. We considered both participants with a healthy, clean oral environment and those with an altered oral environment resulting from a broad spectrum of factors, including dental restorations, denture wearing, mucosal changes, and retention of impacted teeth. The findings underscore the significance of the diverse oral conditions under consideration. The decision to use unstimulated whole saliva over stimulated saliva was made to ensure the accuracy of the results, as stimulated saliva may be diluted and, therefore, reduce CFUs. The whole saliva interacts with all oral cavity surfaces, so meticulous care was taken during candidate selection to ensure an unbiased representation of various groups. So, participants with overlapping findings between the two groups were excluded. This rigorous selection protocol affected the sample size in the individual groups. The authors recommend larger-scale studies to verify this study's findings. We also recommend investigating possible changes in the oral microbiome due to various dental and nonpathological mucosal changes, as they might affect the oral tissue's health or the individual's general well-being. Additionally, in the current study, chromogenic plaque was used for the presumptive identification of *Candida* at the species level, as it provides a reported robust sensitivity and specificity for *C. albicans* . However, for atypical candidal strains, more precise methods are recommended, such as the germ tube test, assessment of colony morphology on SDA, cornmeal–Tween 80 agar, conventional biochemical assays, and molecular or genetic approaches like PCR or DNA sequencing. Finally, although the current study controlled for several key variables through statistical models and exclusion criteria, a notable limitation is the lack of detailed data on participants' dietary habits, particularly their consumption of high-sugar meals. Since diet may modulate the oral microbiome and candidal carriage, it should be considered in future investigations for a more comprehensive assessment of such a confounder.

This study identifies patients with fixed restorations as potentially high-risk groups for elevated salivary candidal load. Furthermore, restorative materials should be selected based on their potential to promote microbial adhesion and biofilm formation. NAC colonization is associated with partially erupted third molars, suggesting that prophylactic extraction decisions, particularly in immunocompromised patients to eliminate this microbial reservoir. However, such a decision requires further larger and longitudinal studies before it can be adopted as an option for the prophylactic removal of partially impacted third molars in the healthy population. It appears that the outcome of this study suggests a paradigm shift toward a preventive approach for managing oral candidal ecology, even when an individual is otherwise healthy. Finally, despite conflicting published reports, in patients with idiopathic oral symptoms such as glossodynia or burning mouth symptoms, the presence of these risk factors should prompt further investigation into a high fungal load as a possible etiology.

## Conclusion

This study emphasizes the factors affecting salivary candidal load and highlights the importance of considering both clinical and demographic aspects in managing candidal colonization. As the results indicated, NAC was detected in two-thirds of samples, with minimal variation between groups. *C. albicans* , however, was identified in half of the samples, occurring most frequently in Group 2 (73.7%). *C. albicans* predominated in Group 2, while NAC load (CFUs/mL) was notably higher in participants with impacted molars. Recognizing the colonization patterns in these individuals can aid in selecting restorative materials and preventive measures to reduce colonization and excessive salivary carriage. Further research is needed to explore the long-term implications of these findings on oral and systemic health, and to clarify the mechanisms underlying these differences, particularly in restorative materials and oral hygiene practices, to optimize the management and prevention of candidal infections in vulnerable populations.
